# A Review of Psychological Issues among Patients and Healthcare Staff during Two Major Coronavirus Disease Outbreaks in China: Contributory Factors and Management Strategies

**DOI:** 10.3390/ijerph17186673

**Published:** 2020-09-14

**Authors:** Ka Ming Chow, Bernard M.H. Law, Marques S.N. Ng, Dorothy N.S. Chan, Winnie K.W. So, Cho Lee Wong, Carmen W.H. Chan

**Affiliations:** The Nethersole School of Nursing, Faculty of Medicine, The Chinese University of Hong Kong, Hong Kong, China; kmchow@cuhk.edu.hk (K.M.C.); bernardlaw@cuhk.edu.hk (B.M.H.L.); marquesng@cuhk.edu.hk (M.S.N.N.); winnieso@cuhk.edu.hk (W.K.W.S); jojowong@cuhk.edu.hk (C.L.W.); whchan@cuhk.edu.hk (C.W.H.C.)

**Keywords:** psychological, patients, healthcare staff, coronavirus disease

## Abstract

Outbreaks of severe acute respiratory syndrome (SARS) and coronavirus disease 2019 (COVID-19) have affected populations worldwide. Our literature review summarises the studies reporting psychological issues among healthcare staff and infected patients in mainland China, Hong Kong, and Taiwan during these two outbreaks and the potential strategies for addressing these issues. Our review shows that patients and healthcare staff presented similar psychological symptoms, including anxiety, fear, distress, and depression, which may lead to stress-related complications such as insomnia. In patients, these psychological impairments can be contributed to by being quarantined, perceptions of threats to life, and uncertainty about health status. Quarantine is also a factor for distress among healthcare staff, together with their heavy workload, the fear that they and their families would become infected, witnessing their patients’ poor and deteriorating conditions, and the requirement to wear protective gear. Strategies that are needed to address these factors include providing counselling services, implementing mindfulness-based therapies and optimism interventions, and providing telecommunication facilities for patients to communicate with their families. Healthcare staff should also be provided with these services, together with appropriate and flexible work shift arrangements and morale boosting. These strategies would improve not only the mental well-being of patients and healthcare staff, but also the self-efficacy and competence of the staff to provide quality healthcare services.

## 1. Introduction

Epidemics and pandemics of potentially fatal infectious diseases are generally considered to be public health disasters that negatively affect public health and people’s lives in a multi-faceted manner [1,2]. To prevent the spread of such disease outbreaks, patients generally have to undergo compulsory quarantine, which has negative effects on their psychological and mental health, as shown by multiple studies worldwide [3]. Likewise, healthcare staff such as nurses, doctors, and medical assistants need to work overtime and under stress during such epidemics and pandemics to manage the increasing number of infected patients. They may experience emotional difficulties through witnessing the loss of their patients’ lives and through their perceived constant risk of infection [4]. All of these factors could contribute to the development of psychological and mental health issues. These issues not only further deteriorate the health status and quality of life of patients, but also limit the ability of healthcare staff to deliver quality care and treatment to the patients for their rehabilitation [5] and even cause tension between the patients and healthcare providers [6]. To enhance the mental well-being of both the patients and healthcare staff, strategies are warranted to address psychological issues during disease outbreaks. To achieve this, a better understanding of the extent of the effect of such epidemics and pandemics on the psychological health of these individuals and the factors that contribute to its deterioration is needed.

Severe acute respiratory syndrome (SARS) and coronavirus disease 2019 (COVID-19) are two major coronavirus disease outbreaks with epicentres in China, the country with the largest population on earth. Both diseases are caused by highly contagious strains of pneumonia-triggering SARS-coronaviruses. Cases of super-spreading events of SARS, where one individual is capable of infecting more people than expected, were reported in China [7] and such events may have occurred during the COVID-19 pandemic as well [8]. Both outbreaks resulted in a considerable number of confirmed cases and deaths both nationally and worldwide. During the 8-month SARS epidemic during 2002–2003, 8,096 SARS cases were reported worldwide, with a death toll of 774. China was the country most affected by the SARS epidemic, with 92% and 89% of the confirmed SARS cases and SARS-related deaths worldwide, respectively, being reported in mainland China, Hong Kong, Macao, and Taiwan [9]. The COVID-19 pandemic is ongoing and the numbers of confirmed cases and COVID-19-associated deaths are rising daily. As of early May 2020, COVID-19 has affected more than 3.1 million people worldwide and has resulted in more than 224,000 deaths [10]. In China, more than 84,000 people have been infected and more than 4600 COVID-19-associated deaths have been reported [10]. Notably, the coronavirus responsible for this pandemic has a high rate of transmissibility, a feature that enables the COVID-19-causing virus to infect more than 80,000 people in China within two months [11]. Despite the severity of the coronavirus disease outbreaks, little is known about the causes of SARS and COVID-19, as well as the mode of transmission of these viruses, given the novelty of the coronavirus strains at the time of the respective coronavirus outbreak. Consequently, there was little knowledge about the prevention and treatment of the diseases at the time of their outbreaks. All of these factors have contributed to the increased level of perceived susceptibility of the public to coronavirus diseases, especially the healthcare staff who manage infected patients.

Given that SARS and COVID-19 have affected a large number of civilians in China, it is tempting to speculate that patients and healthcare staff were under considerable psychological pressure regarding their illness and caring for the fast-growing number of patients, respectively. This prompts a need for a closer examination of the psychological status of these Chinese patients and healthcare staff and the causes of their psychological issues to not only enable the development of effective strategies to reduce their psychological stress, but also to better prepare them for another potential epidemic or pandemic in the future. Here, we review previous studies that reported the occurrence of psychological symptoms, including anxiety, depression, fear, stress, and stress-induced complications, among Chinese patients and healthcare staff during the SARS and COVID-19 outbreaks in China. The review aims to identify the factors that contribute to the occurrence of these psychological symptoms and the ways to effectively address the psychological issues in order to provide healthcare providers and healthcare policy makers in China with pertinent information to develop strategies for enhancing the psychological well-being of both the patients and healthcare staff during the present and future epidemics or pandemics.

## 2. Methodology of the Literature Review and Overall Results of the Literature Search

Our literature search was conducted in May 2020. We reviewed and included articles published in English or Chinese, which we extracted from PubMed and Wanfang databases. We included original studies and reviews that reported psychological issues, such as depression, anxiety, post-traumatic stress disorder (PTSD), fear, somatisation, and obsessive-compulsive disorder, of Chinese SARS or COVID-19 patients and Chinese healthcare staff caring for those patients. Studies that identified factors leading to the psychological issues among these patients and healthcare staff were also included. As the scope of our review was the psychological issues among Chinese patients and healthcare staff, studies involving sample populations of non-Chinese patients and healthcare staff were excluded. The search strategy used for this review is presented in Table 1.

After the literature search, we had obtained a total of 50 original articles that either reported the occurrence of the psychological symptoms of Chinese patients or Chinese healthcare staff during SARS and COVID-19 outbreaks in China, the contributory factors to these issues, or provided recommendations on the strategies to address these issues. Of these, 25 articles (50%) were published in Chinese. Thirty articles reported such psychological issues during the SARS outbreak, of which 15 (50%) were published in Chinese. The remaining 20 studies reported the aforementioned issues during the COVID-19 pandemic, with 11 of these (55%) being published in Chinese. Moreover, 30 studies reported the occurrence of the psychological issues among the aforementioned subjects during the two disease outbreaks. The demographic characteristics of the participants in these studies, the major outcomes of interest, and the instruments used for assessing these outcomes are presented in Supplementary Tables S1−S4.

## 3. Psychological Issues of SARS Patients during the SARS Epidemic

The SARS epidemic exerted a negative psychological impact on patients suffering from the disease. SARS patients were reported to exhibit significantly increased stress levels due to worry about their physical health and potential financial stress [12]. As a result, they experienced symptoms such as insomnia, depression, and a reduced social life [12]. In another study conducted in Hong Kong [13], almost 60% of SARS survivors had developed psychiatric disorders during their recovery and almost half of these individuals had symptoms or disorders such as depression or PTSD, with PTSD being diagnosed using the clinical criteria from the Diagnostic and Statistical Manual-IV (DSM-IV). Notably, some of these survivors developed other complications such as agoraphobia, panic disorder, and social anxiety disorder [13], which further deteriorated their psychosocial and psychological well-being.

Notably, some patients continued to exhibit psychological impairments after their recovery from SARS. Studies demonstrated that 13% to 18% of SARS survivors in Hong Kong continued to suffer from anxiety and depression for one to three months after recovery [14,15]. Nevertheless, the severity of their PTSD symptoms was found to decrease during the recovery process [14]. In another study [16], a significant proportion of Chinese SARS survivors (44%) were diagnosed with PTSD using another clinical criteria, named Chinese Classification of Mental Disorders Version 3 (CCMD-III), within four years after treatment. Moreover, most patients (82%) diagnosed with PTSD at the time of hospital discharge continued to suffer from PTSD for the next four years. These findings suggest that the psychological impact of SARS on patients is likely to be long-term.

## 4. Psychological Issues of COVID-19 Patients during the COVID-19 Pandemic

Although limited studies have examined the psychological status of Chinese COVID-19 patients in the ongoing pandemic, a few studies have provided evidence that COVID-19 patients generally suffer from psychological symptoms similar to those reported in SARS patients and survivors. Evidence shows that these patients experience increased anxiety and depression [17,18,19,20]. One study showed that more than one third of the Chinese COVID-19 patients have developed moderate to severe levels of anxiety [17]. Another study also showed that most of the studied COVID-19 patients (96%) have developed PTSD, although it should be highlighted that the assessment of PTSD symptoms in the study relied primarily on self-report rather than clinical diagnosis [21]. Of note, however, there has also been an update of the clinical criteria used for diagnosis of PTSD in 2013, where the Diagnostic and Statistical Manual of Mental Disorders, the criteria for assessing posttraumatic stress symptoms, was modified [22]. Owing to such modifications and the use of different methodologies for PTSD diagnosis among patients between the studies during the two different coronavirus disease outbreaks [13,16,21], it is important to note that the effect of these outbreaks on the prevalence of PTSD among patients cannot be directly compared. Moreover, these patients were shown to have a perception of helplessness and a tendency to resign to their fate during their rehabilitation [23], which promoted a sense of frustration among these individuals.

## 5. Psychological Issues of Healthcare Staff during the SARS Epidemic

Multiple lines of evidence suggest that Chinese healthcare staff experienced multiple psychological symptoms because of their daily duties of caring for SARS patients. Stress was found to be one of the most common psychological issues among nurses during the SARS epidemic. In Hong Kong, 68% to 80% of nurses often experienced stress during the SARS epidemic and most of them perceived that their stress was work-related [24]. In a study conducted in mainland China, more than 60% of the health workers had perceived stress and fear and some of them suffered from sleep difficulties and nervous breakdown because of this stress [25]. A significant proportion (10% to 40%) of hospital staff, including nurses and medical staff, were also found to develop anxiety, depression, or PTSD [26,27,28,29]. Moreover, based on the DSM-IV criteria, one study found that 5% of healthcare staff, including doctors, nurses, and physician assistants, developed acute stress disorder [30]. Younger and female nurses [28,29] and nurses working in SARS wards [26] were more susceptible to developing a higher level of depression. Interestingly, nurses were reported to be more susceptible to the experience of more severe depression compared to doctors, as indicated by the significantly higher proportion of nurses reporting at least a moderate level of depression than doctors in Chinese hospitals during the SARS epidemic [31]. A significantly higher level of depression severity was also observed among these nurses. Similarly, disparities in the propensity to experience depression were observed between frontline and second-line doctors and nurses as significantly more frontline doctors and nurses were found to have at least a moderate level of depression than the second-line counterparts [31]. Nevertheless, one study reported that the anxiety and depression experienced by nurses during their early days of working in SARS wards reduced significantly after several weeks [32].

Several research groups implemented the Symptom Checklist 90 (SCL-90) to demonstrate the multiple aspects of psychological impairments suffered by Chinese healthcare staff during the epidemic. The SCL-90 is an instrument for assessing the severity of nine psychological issues, including somatisation, depression, and anxiety. In multiple studies, Chinese healthcare staff were reported to exhibit increased severity of various psychological issues examined in the SCL-90 [5,33,34,35]. For example, during the early weeks of being deployed to SARS wards, hospital staff not only suffered from increased anxiety and depression, but also experienced higher levels of somatisation, i.e., they exhibited physical symptoms associated with higher stress, such as headaches, increased blood pressure, and sleep difficulties [34]. Nurses were also found to exhibit some obsessive-compulsive symptoms such as feeling that their hands are still dirty after washing them multiple times [36]. Another notable finding was that Chinese nurses, especially those working in the frontline, tended to be more stressed and reported a significantly higher SCL-90 score compared to other types of hospital staff such as doctors and health assistants. This is likely because the responsibility of providing intensive care to the SARS patients fell on nurses, which subjected them to a heavy workload and amplified their worry about being infected due to their close contact with the infected patients. Overall, the SARS outbreak had a multi-faceted negative impact on Chinese healthcare staff, especially nurses who were in close contact with SARS patients.

## 6. Psychological Issues of Healthcare Staff during the COVID-19 Pandemic

Examination of the prevalence of psychological symptoms among Chinese healthcare staff during the COVID-19 pandemic yielded similar findings to those during the SARS epidemic, with anxiety, depression, and stress being the common symptoms [37,38]. Healthcare workers in Chinese hospitals have been shown to have a higher incidence of insomnia, anxiety, depression, and obsessive-compulsive disorder compared to individuals working in a non-medical setting [39,40], highlighting the pressure caused by healthcare job demands. Consistent with this, one study also showed that nurses exhibit a significantly higher level of anxiety compared to the reported national standard level of anxiety among nurses before the pandemic [41]. Notably, healthcare staff who work in the frontline appear to be more susceptible to higher levels of psychological symptoms [37,42], potentially due to their need to be in frequent contact with the patients and their heavy workload. However, unlike the findings for patients, there is currently no evidence that healthcare staff suffered from PTSD during the COVID-19 outbreak.

Interestingly, despite the experience of psychological stress and high work pressure, nurses in two qualitative studies were reported to possess a sense of mission and responsibility in delivering quality nursing care to patients [43,44]. Instead of feeling intimidated by the severity of the pandemic, these nurses considered their duty in patient care as a way to contribute to society. Some nurses even felt a sense of accomplishment when recovered patients expressed their gratitude to them for providing intensive care during rehabilitation [44]. Thus, these findings demonstrate that healthcare staff could have both positive and negative experiences in their role of providing patient care and therefore, encouraging them to focus on the positive aspects of their role may help reduce psychological issues during such outbreaks.

## 7. Factors contributing to Psychological Issues among Patients during the Coronavirus Disease Outbreaks

Several association and qualitative studies have examined the causes of psychological impairments among Chinese SARS and COVID-19 patients. These causes include perceived life threats, being quarantined, and uncertainty about the health status.

### 7.1. Perceived Threats to Life

Patients’ fear of the health-related consequences of infection was suggested to be a factor contributing to their distress [19]. Given the novelty of the coronavirus diseases at the time of their outbreaks, medical professionals had little knowledge about the treatment of these diseases. During the outbreaks, no effective medications or vaccines were available for disease treatment and hospitalised patients were only given treatment for symptom management [45]. Given the increasing number of deaths from the disease, patients would likely perceive that their illness may not be successfully cured and that they may eventually die. Such a perception would trigger psychological distress. Indeed, perceived life threats were shown to be associated with depression and to be strong predictors of anxiety among Chinese SARS survivors in Hong Kong [14,15], although no studies have shown that perceived life threats are associated with Chinese COVID-19 patients as of May 2020.

### 7.2. Being Quarantined

During the SARS and COVID-19 outbreaks, patients were required to undergo mandatory quarantine in hospital in an attempt to prevent super-spreading events. During such quarantine periods, visits from families and friends were forbidden. The resulting separation from their loved ones and social isolation were suggested in a case report study to be potential factors for the development of psychiatric problems, including anxiety and depression, among the patients, where such isolation had led to an exacerbation of depressive symptoms among patients [46]. Moreover, mandatory quarantine may induce boredom and loneliness [12], which could be caused by restrictions in the opportunities to communicate with their families. This would likely increase their susceptibility to developing psychological symptoms such as depression, stress, and anxiety. In a study conducted among SARS patients [12], these patients expressed feelings of boredom and loneliness and a modest proportion of them (over 20%) had a depressed mood. Indeed, loneliness as a result of social isolation during a coronavirus disease outbreak was previously suggested to be a risk factor for a repertoire of psychological symptoms including depression, anxiety, and stress [47]. In contrast, SARS patients who have more opportunities to talk to and share their worries with others were shown to be less depressed [15].

### 7.3. Uncertainty about Health Status

Patients’ worries about their health conditions during their treatment may also have contributed to their stress. One study [18] revealed that COVID-19 patients generally have feelings of uncertainty throughout the treatment process at hospital. They were reported to possess a feeling of uncertainty regarding the changes in the health status as they were not informed of the causes of these changes. Such uncertainty was also shown to be associated with patients’ fear. This would explain the finding demonstrating that a considerably higher proportion of COVID-19 patients were found to have developed depression and anxiety symptoms compared to healthy controls [18]. Moreover, COVID-19 patients were also suggested to have little confidence in the prospect of being cured [23], which could potentially exacerbate the feeling of uncertainty of the deterioration in their physical status during treatment, owing to the lack of confidence on the effectiveness of the treatment received. They possessed little knowledge of the disease and reported that they received inadequate information on the changes in their health status. They were therefore uncertain and worried about the extent to which their health conditions had deteriorated due to the illness [18]. The perception of such uncertainties would lead to increased stress among the patients. Likewise, SARS patients were also shown in a case report study to develop psychiatric symptoms such as anxiety, depression, and anger owing to the severity of the symptoms they experienced [46], adding further weight to the observation that the feeling of uncertainty of the patients regarding their health status contributes to psychological issues.

## 8. Factors Contributing to Psychological Issues among Healthcare Staff during the Coronavirus Disease Outbreaks

The causes of psychological issues among healthcare staff during the SARS and COVID-19 outbreaks have been studied extensively. Overall, six main causative factors have been identified, namely heavy workload, concerns about patients’ conditions, requirement of wearing protective gear for long hours, being quarantined, fear of being infected, and concerns about their families being infected.

### 8.1. Heavy Workload

Nurses, doctors, and hospital staff face huge pressure when performing their duties in patient care and treatment. Several studies have suggested heavy workload to be one of the major factors causing stress and psychological issues among these individuals during both the SARS and COVID-19 outbreaks [41,42,48,49]. Such a heavy workload was likely impacted by the rapid increase in patient numbers during the coronavirus outbreaks and a manpower shortage [25,50,51], which increased the job pressure. Indeed, in one study, the majority of medical workers (85%) expressed that job pressure was high and some of them believed that they were not able to bear the pressure [52]. The increasing workload could lead to exhaustion of the frontline healthcare staff, increasing their experience of fatigue and anxiety. In a previous study after the SARS outbreak, the anxiety levels of healthcare staff were shown to be significantly higher than those of administrative staff in hospitals and their anxiety levels were demonstrated to be positively associated with their burnout levels [48]. Likewise, nurses who provided care for SARS patients were found to exhibit depressive symptoms due to their perceived difficulties in bearing the heavy workload [29] and they tended to develop somatisation disorder, experiencing physical symptoms such as headache, sleep difficulties, and high blood pressure [34]. Consistent with these findings, in another study, nurses caring for COVID-19 patients expressed a need for more manpower to work in the frontline healthcare duties [53].

### 8.2. Concerns about Patients’ Conditions and Feeling Helplessness in Quality Care Provision

Witnessing the deteriorating condition of patients is also a work-related source of distress among Chinese healthcare staff, particularly nurses, during patient care delivery. In one study, a significant proportion of nurses (61% to 89%) expressed that while caring for SARS patients, witnessing their sufferings made them feel sad and also guilty if they could not provide the patients with what they needed [54]. Consistent with this finding, frontline nurses in the COVID-19 pandemic have been reported to frequently exhibit frustration on witnessing the poor condition and anxiety of their patients [43]. Under such circumstances, nurses may develop a feeling of “powerlessness” in providing care to the patients, in turn leading to psychological distress [55]. Such feelings are more prominent among younger nurses, who have less experience in frontline duties during disease outbreaks such as SARS [29]. Based on this finding, it is tempting to speculate that nurses may not have the confidence that they would be able to get over the adversity of SARS and COVID-19 outbreaks. Feelings of helplessness in healthcare staff may also be exacerbated by their lack of a complete understanding of the coronavirus disease they are facing. For example, Zhou et al. suggested that limited knowledge of healthcare staff on COVID-19 would contribute to their loss of confidence in the prospect of effectively combating the outbreak [49]. Consistent with this finding, a study reporting the perception of a nurse manager in Hong Kong also demonstrated that nurses’ limited knowledge of SARS and their perceived uncertainty about the status of the disease outbreak had contributed to their anxiety, anger, and sense of helplessness [56]. The circulation of rumours during the SARS epidemic was also reported to have contributed to such perception of uncertainty caused by their lack of knowledge of SARS [55]. In sum, healthcare staff may experience stress through witnessing patients’ poor conditions, which may make them feel helpless in providing quality care to their patients. This helplessness may be aggravated by the limited knowledge of the staff on how to effectively combat the coronavirus disease outbreak they are facing.

### 8.3. Requirement to Wear Protective Gear

A few studies have demonstrated that the need to wear protective gear during their duties could also contribute to stress among healthcare staff. As the protective gear fully covers the body, it causes significant physical discomfort, such as the sensation of overheating. Several studies in mainland China and Taiwan have reported such discomfort to be a source of stress and difficulty among nurses during both the SARS and COVID-19 outbreaks [25,43,51,57,58]. Discomfort due to protective gear was also demonstrated to be positively associated with anxiety levels among healthcare staff in hospitals [48]. Nevertheless, a study showed that inadequate supply of protective gears during the COVID-19 outbreaks was also a causative factor for psychological issues, including anxiety, depression, and fear, among healthcare staff [39].

### 8.4. Being Quarantined

Healthcare staff in China deployed to care for coronavirus disease patients were required to work in isolation wards and to undergo self-isolation when they were off-duty, during both the SARS and COVID-19 outbreaks [31,44]. Like patients, these healthcare staff were shown to exhibit various psychological issues due to such isolation. These quarantined individuals were shown to be more likely to develop acute stress disorder [30] or post-traumatic stress symptoms [27]. They also tended to develop depressive symptoms, potentially due to the limited opportunities to express their negative emotions to others [28]. These psychological impairments could also be impacted by experiencing boredom and loneliness and missing their families during quarantine, with limited opportunities to communicate with them, as reported by multiple studies of healthcare staff during the SARS and COVID-19 outbreaks [29,31,41,44,59]. Notably, the mandatory quarantine of healthcare staff would also deprive them of the opportunities to take care of their families [60], which is likely to make them feel anxious about the health of their loved ones.

The need for quarantine of healthcare staff would also likely prevent them from getting the required support from their families. Of note, one of the studies [31] conducted a stepwise regression analysis, examining the association between certain factors and the level of depression indicated by the Zung Self-rating depression scale (SDS) score. The authors reported that the lack of support and interactions with family was positively associated with the SDS score for both frontline doctors and frontline nurses, although a lack of support from society was additionally found to be associated with the SDS score among frontline nurses only. This finding also suggests the bi-directional detrimental effect of quarantine and self-isolation among healthcare staff, where they on one hand would not be able to take care of and communicate with their families and on the other hand, would be unable to get the spiritual support they need for fulfilling their duties.

### 8.5. Fear of Being Infected

Working in isolation wards also raises concerns among healthcare staff about being infected by their patients. During the SARS epidemic, almost half of the infected individuals were healthcare workers [61]. SARS- and COVID-19-causing coronavirus strains have a long incubation period of 2 to 14 days [62], during which infected persons may appear asymptomatic; thus, the lack of symptoms among healthcare staff during their early days of working in isolation wards would merely give them a false sense of security of being uninfected. This suggests that healthcare staff perceive a greater risk of infection when working in these wards. Multiple Chinese studies have reported fear of being infected and worries of passing the infection to others as a source of stress among healthcare staff during both the SARS and COVID-19 outbreaks, which triggers their anxiety and depression [25,39,51,63]. This can be further illustrated by the finding that during the COVID-19 outbreak, nurses who are in frequent contact with patients exhibited a higher anxiety level than doctors about being infected [64]. Being at risk of contracting COVID-19 was also shown to be a risk factor for psychological symptoms, obsessive-compulsive symptoms, and sleep difficulties among healthcare workers [40]. All of these findings suggest that healthcare staff are vulnerable to psychological issues and other stress-related symptoms, owing to their duty of providing care to patients of coronavirus diseases.

### 8.6. Concerns about Families Being Infected

Multiple studies have shown that the healthcare staff in China exhibited anxiety and/or stress due to the fear that their loved ones may contract SARS or COVID-19 [60,63,65,66]. Furthermore, in a study conducted in Taiwan, almost 90% of nurses expressed that their worry about whether their families have contracted SARS was a contributor to their stress [57]. This suggests that concerns about the health of their family members is one of the primary stressors among Chinese healthcare staff. This is not surprising as Chinese culture and teachings often emphasise the value of family and promote the importance of bonding and unity between family members [67]. Therefore, even when healthcare staff are on-duty, they may still feel anxious about the health status of their family members during the coronavirus disease outbreaks and this could in turn affect the quality of care they provide to patients.

## 9. Strategies for addressing Psychological Issues among Patients and Healthcare workers during Coronavirus Disease Outbreaks

Based on the above-mentioned factors that contribute to psychological issues among patients and healthcare staff alike, several studies have provided recommendations on potential strategies to improve the psychological health of these individuals. These strategies are summarised below.

### 9.1. Strategies for Patients

Counselling services should be provided to patients to address their psychological symptoms. These counselling sessions should be conducted by mental health professionals, who can provide expert advice to the patients on how to address their stress. This is in line with the recommendation that mental health professionals should form a part of the workforce managing coronavirus outbreaks [13]. These sessions may be held in the form of video conferences using online communication software, such as Skype and FaceTime, to avoid putting the mental health professionals at risk of infection. These professionals should also proactively monitor the psychiatric symptoms of the patients [46] so that further follow-up actions to address such symptoms can be taken promptly whenever necessary.

However, merely addressing the psychological symptoms of these patients is inadequate. Strategies are also needed to address the factors shown to contribute to these symptoms, such as perceived life threats, loneliness due to quarantine and limited opportunities to communicate with families, and perceived uncertainty about health conditions during treatment. In particular, in an attempt to keep the patients optimistic, nurses who are in close contact with them should develop strategies [23] or proactively explain to them that they will eventually recover. Nurses and mental health professionals should make a collaborative effort in implementing certain optimism interventions for the patients that enhance their understanding of the strategies aimed at relieving stress, maintaining their optimism and keeping a positive attitude towards life, while maintaining physical health by following a healthy diet and doing adequate physical activity. Studies to test whether such optimism interventions are effective in addressing the psychological issues among patients are recommended. Further, as suggested by Zhou [23], isolation wards that house patients should also be equipped with sufficient telecommunication facilities so that they can communicate with their families via face-to-face video chat. Tablet computers with internet access and Skype installed should be provided to the patients for contacting their families. This would enable them to not only communicate with their loved ones, but to also obtain regular updates on the health status of their families. This would address their feelings of loneliness and missing their families during quarantine in isolation wards. Moreover, social programmes for patients may also be arranged in the isolation wards and they could be encouraged to socialise with other patients [23] so that befriended patients can share their worry or feelings of depression with each other and encourage each other to face the adversity.

### 9.2. Strategies for Healthcare Staff

As multiple factors contribute to psychological issues among healthcare staff, a combination of strategies is warranted to address these issues among these individuals.

Healthcare staff are known to have psychological issues similar to those among patients; therefore, the mental health support suggested for patients should also be provided to the healthcare staff to promote their mental well-being. This is especially important among female frontline staff, owing to their increased risk of anxiety and depression [37]. They should be provided with opportunities to communicate with their families through telecommunication facilities. During off-duty, healthcare staff who experience psychological symptoms should be provided counselling services by mental health professionals. The mental health professionals should not only listen to the staff’s worries, but also offer advice on how to relieve their work-related stress, reinforce their optimistic and positive attitude towards work, and help and encourage them to recall any pleasurable memories of working with patients. Indeed, facing adversity with positivity was demonstrated to be negatively correlated with the experience of somatisation [68] and psychological stress [69] among healthcare staff. To enhance the positive effect of counselling sessions, they can be supplemented with components of relaxation training, which can be delivered in the form of stress relief programmes [70]. The benefits of implementing programmes that include relaxation training, which involves education on techniques for muscle relaxation and diaphragmatic breathing, in reducing stress levels and addressing psychological symptoms among Chinese nurses have also been demonstrated [38,70].

In addition to muscle relaxation and diaphragmatic breathing, the implementation of psychological interventions involving other components such as provision of a hotline for psychological counselling, setting up a group chat room for information sharing between participants, and group-based critical incident stress debriefing (CISD) may also be considered. Group-based CISD involves the relief of stress through the conduction of group conversations with mental health professionals, where participants are given opportunities to express their concerns and worries. Notably, this intervention was shown to be effective in a number of psychological symptoms, such as anxiety, depression, somatisation, and fear, among Chinese healthcare staff stationed in intensive care units, as shown by the considerable decrease in the proportion of participants experiencing these symptoms when they were assessed via an author-developed questionnaire [38]. The implementation of such multi-component psychological interventions among healthcare staff caring for coronavirus disease patients should therefore be recommended.

The level of social support received by medical staff was shown to be positively correlated with their sleep quality and self-efficacy in performing their duties and negatively correlated with anxiety and stress [71]. This implies that the perception of being cared for could serve as a driving force for quality care provision by healthcare staff and a barrier to their psychological impairment. Thus, in addition to providing counselling services and psychological interventions, support groups should be formed among healthcare staff [71]. These groups can function as platforms to enable mutual encouragement among the staff, enabling them to perceive the care and concern of their colleagues and managers. For example, WhatsApp groups may be formed, where staff can share their work-related experience with one another, including both the joy of being appreciated by their patients and the stress caused by their heavy workload. Staff can also exchange encouraging messages in the group [72], send best wishes, and motivate one another in combating the coronavirus outbreak. Research developments and updated information on the novel coronavirus may also be shared in the group, which may enhance the staff’s knowledge of the disease [60]. Moreover, informal group chats may be created among healthcare personnel during their off-duty periods, where they can share their concerns with their colleagues; this could be an effective strategy to help release their negative emotions [56].

Given the observation that Chinese healthcare staff may have positive experiences while providing patient care and treatment, encouraging them to focus on the positive aspects of their duties could be an effective approach to reduce their stress levels. To this end, strategies to boost the morale of healthcare staff need to be implemented. Senior management of hospitals should proactively promote the sense of responsibility among the healthcare staff, especially those who work at the frontline, by reminding them that their roles could make an important contribution to public health and their hard work would pay off in the end. When their sense of responsibility is enhanced, healthcare staff may view their duties as a mission and consider their contributions more positively. The reinforced positivity towards their responsibility may in turn help address their psychological issues. Likewise, the strengthening of the team spirit among the healthcare staff would also alleviate their psychological issues by enabling them to realise that they are not fighting the public health crisis alone [56].

To address the contribution of heavy workload to the stress experienced by healthcare staff, hospital management should also provide them with more flexible work arrangements. Appropriate frequency of duty breaks and duration of work shifts should be arranged [57], an approach that may reduce the discomfort experienced by the staff from wearing protective gear for extended periods. Recreational activities may also be organised at the institution [29,41] and off-duty staff should be encouraged to participate in them. Such participation may not only relieve stress among these individuals, but also strengthen the bonding between colleagues, thereby encouraging them to be more supportive of one another.

## 10. Discussion

### 10.1. Implications for Practice

As indicated, several factors may have contributed to the perception of stress among Chinese patients and healthcare staff during coronavirus disease outbreaks, such as being quarantined, fear of being infected, and concern about their loved ones being infected. Such experiences of stress could cause psychological impairments among these individuals. A combination of stress-relieving strategies is required to address such psychological issues. Here, we set out certain strategies that could be implemented for practice for the alleviation of psychological issues among coronavirus disease patients and healthcare staff during the current COVID-19 pandemic and any future outbreaks. As suggested, quarantined individuals should be provided with telecommunication facilities to maintain communication with their loved ones [59], e.g., Skype and other apps that facilitate video calls. At hospitals, additional computing tools with telecommunication apps installed should be provided for rent at no cost to patients and healthcare staff to allow them to contact their families at their convenience. Second, healthcare staff should be provided with opportunities to receive psychological interventions that were shown to have stress-alleviating effects among Chinese patients or healthcare staff, notably during their off-duty time. As indicated above, with research showing that multi-component psychological interventions involving psychological counselling, group-based CISD, and education of relaxation techniques is effective in the relief of a number of psychological symptoms among healthcare staff [38], the incorporation of this intervention in healthcare practice for stress relief among these individuals should be promoted.

Given the known detrimental effects of the past and present coronavirus outbreaks on the well-being of patients and healthcare staff, our preparedness for another novel infectious disease outbreak should be emphasised [73], including preparedness for the negative impact of these outbreaks on the psychological health of healthcare staff [74,75]. Accordingly, more mental health support services should be implemented in preparation for an increased need for psychological support among healthcare staff should another coronavirus disease outbreak strike in the future. As suggested by Lee et al. [57], more staff should be employed in the workforce managing the provision of psychiatric health services. Healthcare staff should also be educated and trained on their ability to exhibit emotional resilience after the end of the COVID-19 pandemic, so as to increase their self-efficacy and competence in handling any psychological issues that they may face during any future health disaster.

### 10.2. Implications for Further Research

This review identified the negative impact of coronavirus outbreaks on the psychological outcomes of Chinese patients and healthcare staff. Notably, most studies have followed a cross-sectional design to investigate psychological outcomes and none have conducted a longitudinal evaluation. Therefore, the long-term impact of coronavirus outbreaks on the psychological health of patients and healthcare staff remains unclear. Therefore, quantitative studies involving repeated measurements of psychological outcomes at different time points using validated instruments are recommended. These studies may also be coupled with qualitative interviews of participants at the final data collection time point to explore the changes in their psychological outcomes or any unmet psychological needs. Notably, previous Chinese studies have focused primarily on the negative psychological experiences of patients and healthcare staff during the coronavirus outbreaks and strategies for their positive adaptation during such outbreaks, such as their approaches to retain positivity in the face of disaster, appear to be understudied [76]. Qualitative studies on this aspect are warranted. Understanding how individuals can positively cope during epidemics or pandemics would provide insights into the development of interventions to help them recover from psychological issues during public health crises.

Moreover, although the included studies in this review suggested a number of strategies in addressing psychological issues among Chinese patients and healthcare staff, studies on the effectiveness of such interventional strategies specifically on Chinese patients and healthcare staff appear to be lacking. Further, with certain interventions such as yoga, cognitive behavioural therapies, and mindfulness-based interventions having shown to be effective in addressing psychological issues such as anxiety and depression [77,78,79,80], research efforts may also be directed towards the evaluation of the effectiveness of such interventions in reducing these psychological symptoms among the aforementioned Chinese subjects. Indeed, more research on the feasibility and effectiveness of these interventional strategies would be required to provide the evidence base in making a case for the development of the suggested strategies for implementation.

### 10.3. Strengths and Limitations

One major strength of our review is the inclusion of articles that were published in Chinese, which were extracted from the Wanfang database. This review would therefore provide healthcare academics with insights on the findings of studies conducted in a Chinese setting, as well as first-hand data on the psychological issues among Chinese patients and healthcare staff during the SARS and COVID-19 outbreaks. However, this review has four limitations that need to be acknowledged. First, the review is primarily focused on psychological issues among Chinese patients and healthcare personnel and the potential strategies for their management presented in this review are based on studies on such Chinese subjects alone. With the potential difference in culture between Chinese and worldwide populations, the findings presented in this review may not be generalisable to other populations in the world. In view of the cultural difference between Chinese and populations worldwide, we suggest further research examining the ethnic differences of the psychological issues reported by patients and healthcare staff during the current COVID-19 pandemic, as well as the contributory factors of these issues. A review examining these differences may be worthwhile. Second, this review primarily reported the psychological outcomes of healthcare personnel based in hospitals. There is a scarcity of literature data on the psychological outcomes of healthcare personnel in other healthcare settings such as private clinics or community health centres, which may limit the comprehensiveness of this review. Third, a number of the studies in this review utilised a self-report methodology for the assessment of the psychological issues among the participants, some of which conducted data collection via author-developed questionnaires. The assessment of these issues was primarily based on the comparison of the participants’ self-rated score and the pre-set cut-off score for each instrument in defining the psychological issues, rather than through clinical diagnosis. Despite the validity of the instruments used, self-report using these instruments is likely to be less reliable than diagnosis using existing clinical criteria in reporting psychological issues among participants. In other words, some of the findings reported in this review that pertain to the proportion of subjects having a particular psychological issue are primarily based on self-perception of the psychological status of the subjects involved, rather than diagnoses of psychological disorders in a clinical sense. Fourth, a number of the included studies in this review did not mention whether the participants had pre-existing psychological issues before data collection and therefore, the reported prevalence of psychological issues in this review is likely to be an over-estimation. In view of the latter two limitations, the findings of this review therefore need to be interpreted with caution.

## 11. Conclusions

Our review demonstrates that experiencing psychological issues such as anxiety, depression, fear, and distress is common among patients and healthcare staff under the influence of coronavirus disease outbreaks such as SARS and COVID-19, as indicated by the considerable proportion of these subjects having these psychological issues during or after these outbreaks. Moreover, disparities in the susceptibility to experience these psychological issues among different types of healthcare staff were noted. For example, healthcare staff were also found to be more susceptible to experiencing these psychological issues compared to non-healthcare staff such as administrative staff at hospitals. Such increased susceptibility to the experience of psychological issues also applies to frontline healthcare staff, as opposed to second line counterparts. These data demonstrate the detrimental effect of the coronavirus disease outbreaks on psychological health among the frontline healthcare workforce.

Psychological issues among Chinese patients and healthcare staff during coronavirus outbreaks are multi-factorial. Thus, multiple strategies need to be implemented to address not only these psychological issues among these individuals, but also the factors that contribute to them. This could help enhance the mental well-being of the patients and healthcare staff, as well as the self-efficacy and competence of the staff to provide quality care services. Patients and healthcare staff should be provided with counselling services, which can be supplemented with additional components such as cognitive behavioural therapy or relaxation training, to keep them optimistic. They should be provided facilities to communicate with their families so as to address their concerns about the health status of their loved ones. Social or recreational programmes should be implemented to enhance peer support. Senior management may also help reduce the stress experienced by the healthcare staff by allowing flexible arrangements for work shifts and promoting a sense of mission during coronavirus outbreaks. We anticipate that the mental well-being of the patients and healthcare staff would be enhanced by implementing these suggested measures. More importantly, the self-efficacy and competence of healthcare staff in providing quality care and treatment services could be increased, which is a pre-requisite for the control of the current and any future coronavirus outbreak.

## Figures and Tables

**Table 1:** The search strategy.

“nurse” OR “nurses” OR “healthcare professionals” OR “health care professionals” OR “patients” OR “physicians” OR “hospital staff”
AND
“stress” OR “distress” OR “stressful” OR “psychological” OR “anxiety” OR “depression” OR “psychiatric”
AND
“severe acute respiratory syndrome” OR “SARS” OR “coronavirus disease” OR “COVID-19”

## Supplementary Materials

The following are available online at https://www.mdpi.com/1660-4601/17/18/6673/s1, Table S1: Characteristics of studies reporting psychological issues among patients during the SARS outbreak, Table S2: Characteristics of studies reporting psychological issues among healthcare staff during the SARS outbreak, Table S3: Characteristics of studies reporting psychological issues among patients during the COVID-19 outbreak, Table S4: Characteristics of studies reporting psychological issues among healthcare staff during the COVID-19 outbreak.
