# Supplementary material for: A Review of Psychological Issues among Patients and Healthcare Staff during Two Major Coronavirus Disease Outbreaks in China: Contributory Factors and Management Strategies
Source: Int J Environ Res Public Health. 2020 Sep 14;17(18):6673. doi: 10.3390/ijerph17186673 (PMC7557771; doi:10.3390/ijerph17186673)
Supplement: Supplementary file 1 [file ijerph-17-06673-s001.pdf]

Table S1. Characteristics of studies reporting psychological issues among patients during the SARS outbreak.

Study / Ref no	Characteristics of participants of interest	Outcomes of interest assessed	Instruments used for assessing psychological issues of interest	Findings reported in this review
Chua et al 2004 [12]	Patients during SARS outbreak<br>***SARS patients***<br>N = 79<br>Age range: 18-60 years<br>Gender: mixed (34% men)<br>Family status: 95% living with family<br><br>***Healthy controls***<br>N = 145<br>Age range: 18-60 years<br>Gender: mixed (41% men)<br>Family status: 90% living with family	• Stress	• Perceived Stress Scale-10	• Mean score of stress (as measured by Perceived Stress Scale-10) among SARS patients (20.0) was significantly higher than healthy individuals (18.0; p<0.04).<br>• A higher proportion of SARS patients reported negative psychological effects compared to healthy controls (91% vs 68%). These negative psychological effects included boredom, loneliness, depressed mood and worry.<br>• The proportion of SARS patients (90%) and healthy controls (88%) reporting positive psychological effects (such as awareness on hygiene and physical state) was comparable.
Mak et al 2009 [13]	SARS survivors<br>N = 90<br>Age: 41.1 ± 12.1 years<br>Gender: mixed (38% men)<br>Family status: 69% married / cohabiting	• PTSD<br>• Anxiety<br>• Depression	• Impact of Event Scale-Revised (Chinese version)<br>• Chinese version of the Structured Clinical Interview for Diagnostic and Statistical Manual-IV (DSM-IV)<br>• Hospital Anxiety and Depression Scale (Chinese version)<br>o Assessment of anxiety and depression was based on the comparison between the participants' self-rated score and the pre-defined cut-off score for each instrument.<br>o Diagnosis of PTSD was based on the clinical criteria DSM-IV.	• The cumulative incidence rate of any psychiatric disorder among SARS survivors 30 months after the SARS outbreak was 58.9%, including depressive disorders (44.0%), PTSD (47.8%), and various anxiety disorders (21.0%) including agoraphobia (6.6%), panic disorder (13.3%) and social anxiety disorder (1.1%).
Wu et al 2005 [14]	SARS survivors<br>N = 131<br>Age: 41.8 ± 14.0 years<br>Gender: mixed (44% men)	• Anxiety<br>• Depression<br>• PTSD	• Impact of Event Scale-Revised (Chinese version)<br>• Hospital Anxiety and Depression Scale (Chinese version)	• At 3 months after discharge, prevalence rates of anxiety, depression, and PTSD among SARS survivors were 14%, 13%, and 5%, respectively.<br>• The score for PTSD symptoms decreased significantly at 3 months

	Family status: Not specified		o Assessment of anxiety and depression was based on the comparison between the participants' self-rated score and the pre-defined cut-off score for each instrument.<br>o Diagnosis of PTSD was based on the clinical criteria DSM-IV.	after discharge, when compared to that at 1 month post-discharge.<br>o Intrusion (as measured by Impact of Event Scale-Revised) 0.91 ± 0.73 vs. 1.12 ± 0.73; p < 0.001<br>o Hyperarousal (as measured by Impact of Event Scale-Revised) 0.85 ± 0.74 vs. 1.05 ± 0.79; p < 0.001<br>o Anxiety (as measured by Hospital Anxiety and Depression Scale) 5.19 ± 4.48 vs. 5.87 ± 3.89; p < 0.05
Wu et al 2005 [15]	SARS survivors<br>N = 195<br>Age: 41.5 ± 14 years<br>Gender: mixed (43% men)<br>Family status: Not specified	• Posttraumatic stress<br>• Anxiety<br>• Depression	• Impact of Event Scale-Revised (Chinese version)<br>• Hospital Anxiety and Depression Scale (Chinese version)<br>o Assessment of anxiety and depression was based on the comparison between the participants' self-rated score and the pre-defined cut-off score for each instrument.	• At 1 month after discharge, among SARS patients, prevalence rates of anxiety, depression, and PTSD were 14%, 18%, and 6%, respectively.
Hong et al 2009 [16]	SARS survivors<br>N = 70<br>Age: 38.5 ± 12.3 years<br>Gender: mixed (33% men)<br>Family status: 67% married	• PTSD<br>• Anxiety<br>• Depression	• Impact of Event Scale (Chinese version)<br>• Zung Self-Rating Anxiety Scale (Chinese version)<br>• Zung Self-Rating Depression Scale (Chinese version)<br>• Symptom Checklist 90 (Chinese version)<br>• Diagnosis of PTSD was based on Chinese Classification of Mental Disorder Version 3 (CCMD-III)	• At 46 months after discharge, 44.1% of patients developed PTSD during the follow-up.<br>• Among those diagnosed with PTSD, a significantly higher level of anxiety, depression and all symptoms assessed in symptom checklist-90 was reported (p ≤ 0.01).

Symptoms assessed in Symptom Checklist-90 include somatization, obsessive–compulsiveness, interpersonal sensitivity, depression, anxiety, hostility, phobic anxiety, paranoid ideation and psychoticism.

Table S2. Characteristics of studies reporting psychological issues among healthcare staff during the SARS outbreak.

Study / Ref no	Characteristics of participants of interest	Outcomes of interest assessed	Instruments used for assessing psychological issues of interest	Findings reported in this review
尹平 et al 2004 [5]	Doctors and nurses<br>N = 41<br>Age: 28 ± 6.6 years<br>Gender: mixed (22% men)<br>Family status: 39% married<br><br>Control individuals<br>N = 1388	• Psychological symptoms assessed in Symptom checklist-90	• Symptom checklist-90 (Chinese version)<br>o Assessment of psychological issues involved in the checklist was based on the comparison between the participants' self-rated score and the pre-defined cut-off score for the instrument.	• During SARS outbreak, healthcare staff had higher levels of somatisation, anxiety, and phobic anxiety compared with control individuals (p < 0.01).<br>• Nurses reported higher levels of somatisation and hostility compared to doctors (p < 0.05).

	Demographic data not reported			
Chan et al 2005 [24]	Nurses<br>N = 1470<br>Age: Not specified (50% aged 18-34 years)<br>Gender: mixed (8% men)<br>Family status: 51% married	• Perceived stress	• SARS Nurses' Survey Questionnaire	• During SARS outbreak, 68.1 – 80.3% of the nurses either always or often perceived stress as a result of the SARS outbreak.<br>• The majority of the nurses perceived the stress from their work (85.9% = 95.6%)
田向阳 et al 2003 [25]	Doctors and nurses<br>N = 545<br>Age: Not specified<br>Gender: mixed (20% men)<br>Family status: 72% married	• Fear<br>• Stress	• Author-developed questionnaire	• During SARS outbreak, 62.8% of the participants reported fear. 61.5% perceived an increased level of stress, and 5.9% found this stress unmanageable.
Su et al 2007 [26]	Nurses<br>N = 102<br>Age: Age of overall sample was not specified<br>Gender: Female only<br>Family status: 74% married	• Anxiety<br>• Depression<br>• PTSD	• Beck depression inventory (BDI)<br>• Spielberger trait anxiety inventory<br>• Chinese version of the Davidson trauma scale (DTS-C)<br>o Assessment of PTSD, anxiety and depression was based on the comparison between the participants' self-rated score and the pre-defined cut-off score for each instrument.	• During SARS outbreak, the overall prevalence of rate of nurses having depressive symptoms was 27.5%. More nurses working in SARS units reported depression compared to those in non-SARS units (38.5% vs 6.7%).<br>• More nurses working in SARS units developed symptomatic PTSD compared to those working in non-SARS units (33% vs 19%).
Wu et al 2009 [27]	Hospital staff including doctors, nurses and administrative staff<br>N = 549<br>Age: Not specified<br>Gender: mixed (24% men)<br>Family status: 84% married	• Posttraumatic stress<br>• Fear for SARS	• Impact of Event Scale-Revised (Chinese version)<br>• Author-developed questionnaire items	• At 3 years after SARS outbreak, 10% of the participants reported high levels of PTSD symptoms. Among them, 40% had persistently high levels during the 3-year period.
刘朝晖 et al 2003 [28]	Healthcare staff (type of healthcare staff not specified)<br>N = 1806<br>Age: 32.6 ± 8.2 years<br>Gender: mixed (21% men)<br>Family status: Not specified	• Depression	• Zung Self-Rating Depression Scale (Chinese version)<br>o Assessment of depression was based on the comparison between the participants' self-rated score and the pre-defined cut-off score for the instrument.	• During SARS outbreak, 37.2% of healthcare staff had developed depression.<br>• The Zung Self-Rating Depression Scale score among female healthcare staff was significantly higher than that among the male counterparts (45.2 ± 10.5 vs 41.7 ± 10.2, $p < 0.01$).
杨莘 et al 2004 [29]	Nurses<br>N = 387<br>Age: 30.7 ± 7.2 years<br>Gender: mixed (0.3% men)<br>Family status: Not specified	• Depression	• Zung Self-Rating Depression Scale (Chinese version)<br>o Assessment of depression was based on the comparison between the participants' self-rated score and the pre-defined	• During SARS outbreak, the prevalence rate of depression was 24.3% among nurses.<br>• The Zung Self-Rating Depression Scale score among younger nurses was significantly higher than that among the older nurses (46.4 ± 11.6 vs 42.4 ± 10.0, $p = 0.016$).

			cut-off score for the instrument.	
Bai et al 2004 [30]	Various healthcare staff including doctors, nurses and physician assistants<br>N = 218<br>Age: 36.9 ± 8.9 years<br>Gender: mixed (34% men)<br>Family status: 65% married	• Anxiety<br>• Depression<br>• Acute stress disorder	• Author-developed questionnaire<br>• Diagnostic and Statistical Manual of Mental Disorders –IV (DSM-IV) criteria was used for diagnosing acute stress disorder	• During SARS outbreak, 5% of healthcare staff developed acute stress disorder.
万云高 et al 2003 [31]	Doctors and nurses<br>N = 1532<br>Age: Not specified<br>Gender: mixed (18% men)<br>Family status: Not specified	• Depression	• Zung Self-rating Depression Scale (Chinese version)<br>o Assessment of depression was based on the comparison between the participants' self-rated score and the pre-defined cut-off score for the instrument	• During SARS outbreak, a significantly higher proportion of nurses were reported to have at least a moderate level of depression compared to doctors ($p = 0.001$).<br>• Moreover, a significantly higher proportion of frontline healthcare staff (doctors and nurses) were reported to have at least a moderate level of depression compared to the second line counterparts ($p = 0.001$).<br>o 14.8% of frontline nurses, 11.5% second line nurses, 6.8% frontline doctors and 3.9% second line doctors developed at least a moderate level of depression.<br>• At both frontline and second line, nurses were reported to have higher level of depression (as measured by the Zung Self-rating Depression Scale score), compared to doctors ($p < 0.001$).<br>o Nurses vs doctors:<br>▪ Frontline: 37.7 ± 8.6 vs 34.2 ± 8.3<br>▪ Second line: 37.2 ± 8.2 vs 32.9 ± 7.6
郭俊花 2003 [32]	Nurses<br>N = 56<br>Age: 29.2 ± 8.2 years<br>Gender: Female only<br>Family status: Not specified	• Depression<br>• Anxiety	• Zung Self-Rating Depression Scale (Chinese version)<br>• Zung Self-Rating Anxiety Scale (Chinese version)<br>o Assessment of anxiety and depression was based on the comparison between the participants' self-rated score and the pre-defined cut-off score for each instrument	• During SARS outbreak, the prevalence rates of anxiety and depression among nurses during the SARS outbreak were 21.4% and 25.0%, respectively, after working for one week in SARS units.<br>• The prevalence rates of anxiety and depression among these nurses decreased to 7.1% and 12.5% respectively, after working for three weeks in SARS units.<br>• Significant decrease in the level of anxiety and depression (as measured by the Zung Self-Rating Anxiety Scale score and Zung Self-Rating Depression Scale score) was observed among

				nurses over the 3-week period of working in SARS units (p < 0.01).
于欣 et al 2003 [33]	Doctors and nurses<br>N = 149<br>Age: 31 ± 8 years<br>Gender: mixed (27% men)<br>Family status: Not specified<br><br>Control individuals<br>N = 52<br>Demographic data not reported	• Psychological symptoms assessed in Symptom checklist-90	• Symptom checklist-90	• During SARS outbreak, healthcare staff working in SARS units reported a higher severity of psychological symptoms assessed in the symptom checklist-90 (as measured by the Symptom checklist-90 overall score) compared with control individuals (28.6 ± 28.2 vs 10.9 ± 14.7, $p < 0.01$).
刘竞 et al 2005 [34]	Medical staff<br>N = 620<br>Age: 30.6 ± 7.2 years<br>Gender: mixed (35% men)<br>Family status: Not specified<br><br>Control individuals<br>N = 1388<br>Demographic data not reported	• Psychological symptoms assessed in Symptom checklist-90	• Symptom checklist-90	• During SARS outbreak, healthcare staff working in SARS units reported higher levels of most psychological symptoms assessed in Symptom checklist-90 than control individuals ($p < 0.05$).<br>• Except for psychoticism, levels of psychological symptoms assessed in Symptom checklist-90 decreased with time ($p < 0.01$).
柳学华 et al 2003 [35]	Nurses<br>N = 101<br>Age: 30 ± 7 years<br>Gender: mixed (11% men)<br>Family status: Not specified<br><br>Control individuals<br>N = 52<br>Age: 28 ± 6 years<br>Gender: mixed (69% men)<br>Family status: Not specified	• Psychological symptoms assessed in Symptom checklist-90,<br>• Anxiety<br>• Depression	• Beck Depression Scale (Chinese version)<br>• State and Trait Anxiety Inventory (Chinese version)<br>• Symptom checklist-90<br>o Criteria for assessing the severity of anxiety and depression was not defined.	• During SARS outbreak, nurses working in SARS units reported higher levels of depression (as measured by the Beck Depression Scale score), compared to control individuals (6.4 ± 6.5 vs 1.4 ± 1.6, $p < 0.01$).<br>• The nurses also reported a higher severity of psychological symptoms assessed in the symptom checklist-90 (as measured by the Symptom checklist-90 overall score) compared with control individuals (30.2 ± 31.4 vs 10.9 ± 14.7, $p < 0.01$).<br>• Interestingly, there was no significant difference between the anxiety level (as measured by the State and Trait Anxiety Inventory score) between nurses and control individuals ($p > 0.05$)<br>o Nurses vs control:<br>▪ State anxiety score: 42.6 ± 6.2 vs 42.9 ± 6.3<br>▪ Trait anxiety score: 41.8 ± 6.9 vs 42.5 ± 7.3
杨爱军 et al 2003 [36]	Various types of healthcare staff (including doctors, nurses, technical staff and health workers)<br>N = 27	• Psychological health	• Author-developed questionnaire	• During SARS outbreak, a large proportion of the participants (88.9%) expressed that they have obsessive-compulsive symptoms such as having a feeling that their hands are still dirty after washing them multiple times.

	Age: ranging between 18-40 years<br>Gender: Female only<br>Family status: 33% married			

Symptoms assessed in Symptom Checklist-90 include somatization, obsessive–compulsiveness, interpersonal sensitivity, depression, anxiety, hostility, phobic anxiety, paranoid ideation and psychoticism.

Table S3. Characteristics of studies reporting psychological issues among patients during the COVID-19 outbreak.

Study / Ref no	Characteristics of participants of interest	Outcomes of interest assessed	Instruments used for assessing psychological issues of interest	Findings reported in this review
程丽 et al 2020 [17]	COVID-19 patients<br>N = 76<br>Age: Not specified<br>Gender: mixed (41% men)<br>Family status: 63% married	• Anxiety	• Zung Self-Rating Anxiety Scale<br>o Assessment of anxiety was based on the comparison of the comparison between the participants' self-rated score and the pre-defined cut-off score for the instrument.	• 34% of the participants (COVID-19 patients) had developed moderate to severe level of anxiety.
Guo et al 2020 [18]	COVID-19 patients<br><br>***COVID-19 Patients***<br>N = 103<br>Age: 42.5 ± 12.5 years<br>Gender: mixed (57% men)<br>Family status: 82% married<br><br>***Non-COVID-19 patients (healthy controls)***<br>N = 103<br>Age: 41.5 ± 13.1 years<br>Gender: mixed (52% men)<br>Family status: 70% married	• Anxiety<br>• Depression<br>• PTSD	• Patient Health Questionnaire, 9-item version (PHQ-9)<br>• Generalized Anxiety Disorder Assessment 7-item version (GAD-7)<br>• Perceived Stress Scale, 10-item version (PSS-10)<br>• PTSD Checklist (PCL-5), measuring PTSD symptoms based on the Diagnostic and Statistical Manual of Mental Disorders–5 (DSM-5) criteria<br>o Assessment of psychological issues was based on the comparison of the comparison between the participants' self-rated score and the pre-defined cut-off score for each instrument.	• A larger proportion of COVID-19 patients were reported to have depression and anxiety compared to healthy controls.<br>o Depression: 60.2% vs 31.1%<br>o Anxiety: 55.3% vs 22.3%<br>• Median scores for depression, anxiety and posttraumatic stress among COVID-19 patients were significantly higher than those for healthy controls. Below shows the median score and the 1st and 3rd quartile score for each psychological issue.<br>o Depression (measured by PHQ-9 score)<br>▪ 5 (3, 8) vs 2 (0, 5), $p < 0.001$<br>o Anxiety (measured by GAD-7 score)<br>▪ 5 (1, 7) vs 0 (0, 4), $p < 0.001$<br>o Posttraumatic stress (measured by PCL-5 score)<br>▪ 8 (4, 14) vs 4 (1, 8.5), $p < 0.001$<br>• No difference was observed for the median score for perceived stress between the patients and healthy controls.<br>o Perceived stress (measured by PSS-10) score)<br>▪ 13 (10, 17) vs 13 (8, 16)
Bo et al 2020 [21]	COVID-19 patients<br>N = 714<br>Age: 50.2 ± 12.9 years	• Posttraumatic stress	• PTSD Checklist<br>• PTSD diagnosis was based on self-report, not diagnosis using a clinical criteria	• The prevalence of significant posttraumatic stress symptoms was reported to be 96.2% (95% confidence interval: 94.8% - 97.6%)

	Gender: mixed (49% men) Family status: 74.2% living with family			

Table S 4. Characteristics of studies reporting psychological issues among healthcare staff during the COVID-19 outbreak.

Study / Ref no	Characteristics of participants of interest	Outcomes of interest assessed	Instruments used for assessing psychological issues of interest	Findings reported in this review
Lai et al 2020 [37]	Doctors and nurses<br>N = 1257<br>Age: Age range not specified<br>Gender: mixed (23% men)<br>Family status: 67% married	• Depression<br>• Anxiety<br>• Distress	• 9-item Patient Health Questionnaire<br>• 7-item Generalized Anxiety Disorder Scale<br>• Impact of Event Scale–Revised<br>○ Assessment of psychological issues was based on the comparison of the comparison between the participants' self-rated score and the pre-defined cut-off score for each instrument.	• A significant proportion of the participants (healthcare workers) have developed at least a mild level of depression (50.4%), anxiety (44.6%), insomnia (34.0%) and distress (71.5%).<br>• A small proportion of the participants developed severe depression (6.2%), anxiety (5.3%), insomnia (1.0%) and distress (10.5%).<br>• Moreover, significantly higher proportion of frontline healthcare workers have developed at least a mild/subthreshold level of the above psychological issues, compared to second line healthcare workers<br>○ ***Depression:***<br>▪ Frontline vs second line: 58.4% vs 44.6% ($p < 0.001$)<br>○ ***Anxiety:***<br>▪ Frontline vs second line: 51.3% vs 39.4% ($p < 0.001$)<br>○ ***Insomnia:***<br>▪ Frontline vs second line: 40.5% vs 29.1% ($p < 0.001$)<br>○ ***Phobic anxiety score:***<br>▪ Frontline vs second line: 76.0% vs 68.0% ($p < 0.001$)
钟燕萍 et al 2020 [38]	Doctors and nurses<br>N = 20<br>Age: ranging between 21-46 years<br>Gender: mixed (30% men)<br>Family status: Not specified	• Psychological status with outcomes including anxiety, fear, and somatisation,	• Author-developed questionnaire	• Participants (doctors and nurses) were subjected to a psychological intervention for assessing the effectiveness of the intervention on addressing their psychological issues. A large proportion of participants (doctors and nurses) expressed that they suffered from anxiety (89.5%) and insomnia (73.7%) prior to the intervention.
Lu et al 2020 [39]	Medical staff and administrative staff at a hospital<br><br>***Medical staff***<br>N = 2042<br>Age: Not specified	• Fear<br>• Anxiety<br>• Depression	• Numeric rating scale<br>• Hamilton Anxiety Scale<br>• Hamilton Depression Scale<br>○ Assessment of psychological issues was	• A higher proportion of the medical staff developed at least a mild level of anxiety and depression, and suffer from at least a mild level of fear compared to administrative staff at the hospital.<br>○ Anxiety: 25.5% vs 18.7% (p = 0.049)

	Gender: mixed (22% men)<br>Family status: 71% married<br><br>***Administrative staff***<br>N = 257<br>Age: Not specified<br>Gender: mixed (25% men)<br>Family status: 71% married		based on the comparison of the comparison between the participants' self-rated score and the pre-defined cut-off score for each instrument.	o Depression: 12.1% vs 8.2% (p = 0.191)<br>o Fear: 70.6% vs 58.4% ($p <$ 0.001)
Zhang et al 2020 [40]	Medical staff (doctors and nurses, and non-medical staff<br><br>***Medical staff***<br>N = 927<br>Age: Not specified<br>Gender: mixed (27% men)<br>Family status: 82% married<br><br>***Non-medical staff***<br>N = 1255<br>Age: Not specified<br>Gender: mixed (42% men)<br>Family status: 82% married	• Anxiety<br>• Depression<br>• Somatisation<br>• Obsessive-compulsive symptom<br>• Phobic anxiety	• Patient Health Questionnaire-4<br>• Symptom Check List-90-revised	• Medical staff rated a higher score for most of the assessed psychological outcomes compared to non-medical staff<br>• ***Anxiety (measured by the General Anxiety Disorder-2 score):***<br>o Medical staff: 1.51 ± 1.28<br>o Non-medical staff: 1.25 ± 1.23<br>o $p < 0.01$<br>• ***Depression (measured by the Patient Health Questionnaire-2 score):***<br>o Medical staff: 1.35 ± 1.37<br>o Non-medical staff: 1.18 ± 1.28<br>o $p = 0.01$<br>• ***Somatisation symptom score:***<br>o Medical staff: 3.46 ± 5.39<br>o Non-medical staff: 3.04 ± 4.46<br>o $p = 0.70$<br>• ***Obsessive-compulsive symptom score:***<br>o Medical staff: 6.02 ± 6.55<br>o Non-medical staff: 4.99 ± 5.50<br>o $p < 0.01$<br>• ***Phobic anxiety score:***<br>o Medical staff: 3.78 ± 4.18<br>o Non-medical staff: 3.68 ± 3.96<br>o $p = 0.87$
Mo et al 2020 [41]	Nurses<br>N = 180<br>Age: 32.7 ± 6.5 years<br>Gender: mixed (10% men)<br>Family status: 49% married	• Anxiety	• Zung Self-rating Anxiety Scale	• The self-rated score of the Zung Self-rating Anxiety Scale among the nurses during the pandemic was significantly higher than the national standard score for anxiety (32.2 ± 7.6 vs 29.8 ± 0.5, $p < 0.001$)
徐明川 et al 2020 [42]	Nurses<br>N = 41<br>Age: 31.3 ± 2.5 years<br>Gender: mixed (10% men)<br>Family status: 80%	• Psychological symptoms assessed in Symptom checklist-90	• Symptom checklist-90	• A large proportion (85.4%) of frontline nurses have developed psychological symptoms in just 2 weeks after being employed to work at frontline.<br>• Among the psychological issues assessed in the symptom checklist-90, hostility, hostility (51.2%), anxiety (39%), interpersonal sensitivity (27%) and somatisation (22%) were particularly prevalent among these frontline nurses.

Symptoms assessed in Symptom Checklist-90 include somatization, obsessive–compulsiveness, interpersonal sensitivity, depression, anxiety, hostility, phobic anxiety, paranoid ideation and psychoticism.
